# The impact of angiogenesis inhibitors on survival of patients with small cell lung cancer

**DOI:** 10.1002/cam4.2462

**Published:** 2019-08-21

**Authors:** Xiaoshun Shi, Xiaoying Dong, Sylvia Young, Allen Menglin Chen, Xiguang Liu, Zhouxia Zheng, Kailing Huang, Di Lu, Siyang Feng, Grant Morahan, Kaican Cai

**Affiliations:** ^1^ Department of Thoracic Surgery, Nanfang Hospital Southern Medical University Guangzhou P. R. China; ^2^ Harry Perkins Institute of Medical Research, Centre for Medical Research University of Western Australia Nedlands WA Australia; ^3^ Mendel Genes Inc Guangzhou China; ^4^ Mendel Genes Inc Manhattan Beach CA USA

**Keywords:** angiogenesis inhibitors, network meta‐analysis, randomized controlled trial, small cell lung cancer, target drugs

## Abstract

**Background:**

Small cell lung cancer (SCLC) is a highly invasive and lethal neuroendocrine tumor. Antiangiogenic drugs have been reported in the treatment of SCLC. We aimed to provide a comprehensive evaluation of the impact of angiogenic inhibitors on SCLC survival using network meta‐analysis.

**Methods:**

The impact of five angiogenesis inhibitors, that is, vandetanib (Van), bevacizumab (Bev), Rh‐endostatin (End), sunitinib (Sun), and thalidomide (Tha), on progression‐free survival (PFS) and overall survival (OS) was evaluated by conducting a network meta‐analysis. RNA sequencing data were downloaded from publicly available databases.

**Results:**

Nine phase II and III randomized controlled trials (RCTs), that involved 1599 participants, that investigated angiogenesis inhibitors in the treatment of SCLC were included in this meta‐analysis. Sun and Bev achieved better PFS than Tha (Bev VS. Tha, HR = 0.88, 95% CI: 0.79‐0.98, Sun VS. Tha, HR = 0.80, 95% CI: 0.65‐1.00). Moreover, Sun and Bev were superior to placebo in terms of PFS (Bev VS. Placebo, HR = 0.89, 95%CI: 0.81‐0.97, Sun VS. Placebo, HR = 0.81, 95% CI: 0.66‐1.00). Based on this study, we found no significant difference of OS of SCLC. The angiogenesis pathway and expression of target genes were globally deactivated in SCLC tissue.

**Conclusion:**

Results of this network meta‐analysis indicate that the PFS outcome of SCLC with Sun or Bev drugs is superior to that of Tha. The improved therapeutic impact of angiogenesis inhibitors on SCLC needs more evidence, such as long‐term observation in clinical trials, to be validated.

## Graphical abstract

In this manuscript, we performed the up‐to‐date comprehensive network meta‐analysis as well as bioinformatic analysis for the angiogenesis inhibitors on the survival of patients with small cell lung cancer in terms of both PFS and PS. Moreover, we found that the overall expression level of angiogenesis inhibitor targets is low expressed in SCLC tissue compared with matched normal tissue which could be the reason for the unideal response.

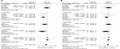

## 1 INTRODUCTION

Small cell lung cancer (SCLC) is a rapidly progressive and easily metastasized pulmonary neuroendocrine tumor, accounting for approximately 15% of lung cancers. In current clinical practice, SCLC is generally treated with chemotherapy combined with radiotherapy. The standard treatment for SCLC is etoposide combined with platinum drugs such as cisplatin or carboplatin.^1^ Patients usually respond well to the drug in the initial treatment, but they quickly develop drug resistance and the disease relapses within 2 years.^2^

The clinical trials of early developed targeted drugs are not ideal and significant effective targeted therapies are needed. In recent years, with a deeper understanding of the pathogenesis of SCLC, a variety of targeted drugs for genetic alteration of SCLC has been developed^3^; these agents include angiogenesis inhibitors, kinase inhibitors, inhibitors of proteases, and immunological checkpoint inhibitors. Among them, angiogenesis inhibitors are currently the most advanced treatment approaches in SCLC clinical research.

It has been well reported that angiogenesis is involved in cancer development in the processes of endothelial cell proliferation, migration, and invasion.^4^ The vascular endothelial growth factor (VEGF) family is the essential antitumor angiogenesis target in both non‐small cell lung cancer and SCLC. VEGF expression levels are variable and are associated with prognosis.^5^, ^6^ Tumor angiogenesis follows multiple steps, including vascular endothelial matrix degradation, endothelial cell migration, endothelial cell proliferation, endothelial cell tube branching to form a vascular ring, and formation of a new basement membrane. Potente et al summarized the role that angiogenesis in cancer development and metastasis and potential therapeutic effects.^7^ Therefore, the inhibition of angiogenesis process could limit or prevent the development and spread of tumor.^8^

Currently, multiple angiogenesis inhibitors have been used for the treatment of cancer,^9^ but no systemic comparison of angiogenesis inhibitors on SCLC has been documented. In this paper, aiming at providing an evidence for the selection of angiogenesis inhibitors, we identify the optimal angiogenesis inhibitors from the treatment of SCLC and potential biological perspective by incorporating network meta‐analysis and bioinformatic analysis.

## 2 METHODS

### 2.1 Data sources

Using three publicly accessible database Cochrane Library (http://www.cochranelibrary.com), Embase (http://www.embase.com) and Pubmed (http://www.ncbi.nlm.nih.gov/pubmed), we systematically searched the published English language literature for reports using angiogenesis inhibitors for the treatment of SCLC published prior to 10 August 2018. Ten angiogenesis inhibitors are included in this study, namely bevacizumab, aflibercept, ramucirumab, sorafenib, sunitinib, nintedanib, pazopanib, vandetanib, cediranib, and endostatin. The keywords that we queried: (a) small cell lung cancer (“small cell lung cancer” or “small cell lung carcinoma” or “small cell cancer of the lung” or “oat cell lung cancer”) and (b) angiogenesis inhibitors (“angiogenesis” OR "angiogenesis inhibitors" OR "targeted therapy" OR "bevacizumab” OR “aflibercept” OR “ramucirumab” OR “sorafenib” OR “sunitinib” OR “nintedanib” OR “pazopanib” OR “vandetanib” OR “cediranib” OR “endostatin” and (c) “randomized controlled study”.

### 2.2 Criteria for inclusion and exclusion of studies

We set up the selection criteria to be included in this meta‐analysis as follows. The study has to be a published English literature on the efficacy of angiogenesis inhibitors in patients with SCLC; and the angiogenesis inhibitors defined as above. The outcome variables must present survival analysis such as progression‐free survival (PFS) and overall survival (OS), meanwhile the statistics such ad hazard rate (HR) and 95% confident interval (CI) must be provided. The design of the included study was randomized controlled study. Only the latest research or the most complete data set was included in final analysis.

Studies with the following characters were excluded from this meta‐analysis. (a) incomplete data that are unable to be used for statistical analysis; (b) comments, letters, reviews; (c) repeatedly used data for multiple studies; (d) study with the number of patients less than 10.

### 2.3 Data extraction and quality assessment

Two investigators of this paper independently extracted the following data, using the same criteria of data extraction and quality assessment, including the name of the first author from the literature, the year of publication, the year of study, the location of study, the research center, the phase of clinical trial, the time of follow‐up in months, the names of angiogenesis inhibitors for each group, the number of patients included in the study, the demographic characteristics (age, sex, and ethnicity), the extent of disease, ECOG performance status, and two indicators of survival analysis, namely the PFS and OS, and corresponding HR and 95% CI value.

For literature quality assessment, randomized controlled studies were assessed using the risk of bias assessment tool suggested by the Cochrane Collaboration Recommendations. If disputes arise in the process of data extraction and quality assessment, a panel discussion was held, and a third investigator was consulted to obtain consistent results.

### 2.4 Bioinformatic analysis

RNA data were acquired from GEO under the accession GSE60052^10^ and reanalyzed by the following bioinformatic pipelines. We first performed an RNA expression differential analysis as previously reported (PMID: 30288103). We then extracted the top 500 significantly downregulated genes to run gene ontology analysis by Functional annotation bioinformatics microarray analysis.^11^, ^12^ The expression level of angiogenesis inhibitor target genes was also extracted for the comparison between SCLC tissue and control tissue.

### 2.5 Statistical analysis

Direct meta‐analysis of two variables was performed by RevMan 5.3. The effect on survival is measured by the value of HR and 95% CI. Prior to data merging, the heterogeneity test was performed on the data of each study. The heterogeneity test was based on Chi‐squared Q test and I^2^ value. If the heterogeneity test had a statistical difference (*P* ≤ .10 or I^2^ ≥ 50%), the random effect model was used to calculate the combined effect value; otherwise, the fixed effect model was used to merge the data (*P* > 0.10 and I^2^ < 50%).

The network meta‐analysis was implemented using "netmeta" package in R version 3.4.3. Using the Cochran's Q‐statistic, the model was selected by the measurement of heterogeneity (if the *P* value of Q‐statistic was greater than 0.05, the fixed effect model was used to combine data; otherwise the random effect model was used).^13^ The intervention measures are ranked according to the *P*‐score—the higher the *P*‐score, the better the survival. The sensitivity analysis of *P*‐score was carried out using random effects and fixed effects models. Publication bias was illustrated by funnel plot.^14^

## 3 RESULTS

### 3.1 Characteristics of the selected studies

**Figure 1:**
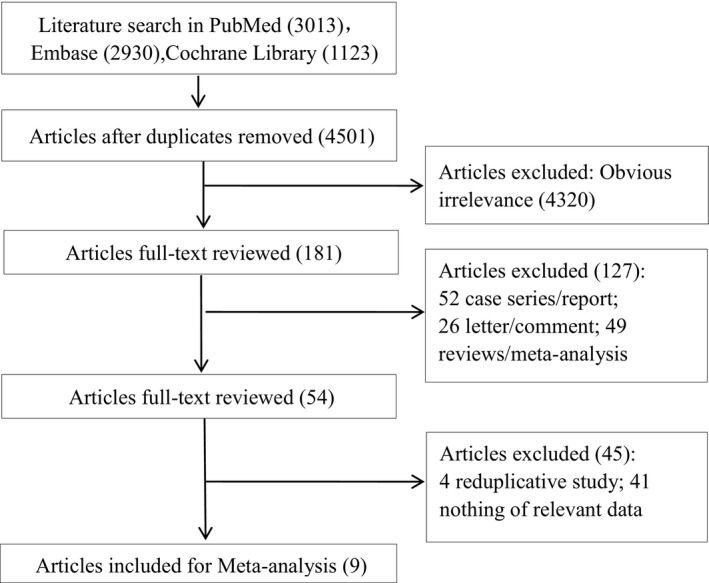
The Flowchart of literature search and study selection. After the workflow of literature review, nine of 7066 literatures were included

As shown in Figure 1, a total of 7066 English publications in PubMed (3013), Embase (2930), and Cochrane Library (1123) were retrieved using the preset search strategy. There were 4501 articles excluded due to being duplicated documents. After browsing the titles and abstracts as well as full‐text review, a further 172 articles, including 52 case series/reports, 26 letters/comments, 49 reviews/meta‐analysis, were excluded because these items are not relevant to the aim of our study. A total of nine qualified studies were retrieved.^15^, ^16^, ^17^, ^18^, ^19^, ^20^, ^21^, ^22^, ^23^

The nine papers were published from 2007 to 2017, with their respective studies starting from 2000 to 2015. The study countries included the United States, China, France, and Italy. Seven reports were of multicenter studies. The clinical trials of SCLC were trial phase II‐III. The median follow‐up time varied from 7.8 to 37.7 months. A total number of 1,599 patients with SCLC were enrolled. These comprised 93 patients in the Vandetanib (Van) group, 190 patients in the Bevacizumab (Bev) group, 69 patients in the Rh‐endostatin (End) group, 44 patients in the Sunitinib (Sun) group, and 414 patients in the Thalidomide (Tha) group. A total number of 789 patients received placebo.

In terms of demographic characteristics, the median age across the studies was in the range of 56.9‐65, and there was no significant difference in age among the treatment groups. As for gender, there were more male patients (981) than female patients (618), but there was no difference between the ratio of male to female between studies. Regarding the extent of disease, most of the patients had extended disease (ED); only two articles reported on both ED and limited disease (LD) with 402 LD patients.^15^, ^16^ In terms of ECOG performance status, patients were mainly distributed in scores 0 or 1, of which 425 and 833 were clearly reported, respectively. The majority of the reported cases were Caucasians from Europe and the United States. One study from China, though did not mention ethnicity, is presumably assigned to Chinese cohort (Table 1). The HR and 95% CI values of PFS and OS were extracted for subsequent survival analysis (Table S1).

**Table 1:** Characteristics of included studies

Author	Public Year	Location	Research Center	Study Year	Trial phase	Follow‐up(months)	Group	Sample size	Median age (range)	Male/female	Extent of disease (ED/LD)	ECOG Performance status (0/1/2)	Race(White/Black)
Arnold AM	2007	USA	NA	2006.4‐2006.6	II	13.5	Van	53	Median: 56.9	27/26	30/23	11/37/5	52/1
							Placebo	54	Median:62.4	31/23	31/23	20/29/5	51/0
Lee SM	2009	UK	multicenter	2003.5‐2006.2	III	37	Tha	365	65(38‐85)	211/154	177/188	54/203/95	NA
							Placebo	359	65(40‐86)	201/158	191/168	69/203/58	NA
Lu S	2015	China	multicenter	2009.7‐2011.8	II	20	End	69	56(40‐76)	56/13	69/0	12/52/5	NA
							Placebo	69	59(36‐73)	57/12	69/0	13/55/1	NA
Pujol JL	2007	France	multicenter	2000.10‐2004.1	III	Minimum time: 36	Tha	49	Median: 59.5	39/10	49/0	20/23/6	NA
							Placebo	43	Median: 59.6	34/9	43/0	16/21/6	NA
Pujol JL	2015	France	multicenter	2009.9‐2011.10	II‐III	37.7 (25‐50)	Bev	37	61.2(43‐75)	25/12	37/0	33(0/1)/3	NA
							Placebo	37	60.1(46‐72)	26/11	37/0	35(0/1)/2	NA
Ready NE	2016	NA	NA	2007.3‐2011.12	II	17.2	Sun	44	59.3(39‐69)	18/26	44/0	20/19/5	41/3
							Placebo	41	60.8(43‐77)	20/21	41/0	17/15/9	40/1
Sanborn RE	2017	USA	multicenter	2008.4‐2013.5	II	18	Van	40	64(47‐74)	24/16	40/0	13/27	34/3
							Placebo	33	63(35‐90)	17/16	33/0	12/21	32/0
Spigel DR	2011	USA	multicenter	2007.3 −2009.2	II	8.1	Bev	52	60(38‐77)	26/26	52/0	15/30/7	47/4
						7.8	Placebo	50	64(47‐82)	30/20	50/0	23/21/6	43/7
Tiseo M	2017	Italy	multicenter	2009.11‐2015.10	III	34.9	Bev	101	64(45‐79)	69/32	101/0	53/42/6	NA
							Placebo	103	63(41‐81)	70/33	103/0	57/35/11	NA

Abbreviations: Bev, Bevacizumab, ECOG, Eastern Cooperative Oncology Group; ED, extensive disease; End, Rh‐Endostatin; LD, limited disease; OS, Overall Survival; PFS, Progression‐free survival; Sun, Sunitinib; Tha, Thalidomide; Van, Vandetanib.

The RCT quality assessment showed that the included literature was of high quality overall. But some of the literature showed a high risk of bias or unclear risk of bias in Allocation Concealment (selection bias) and Blinding of participants and personnel (performance bias), which in other articles was of low risk of bias (Figure S1).

### 3.2 Meta‐analysis

**Figure 2:**
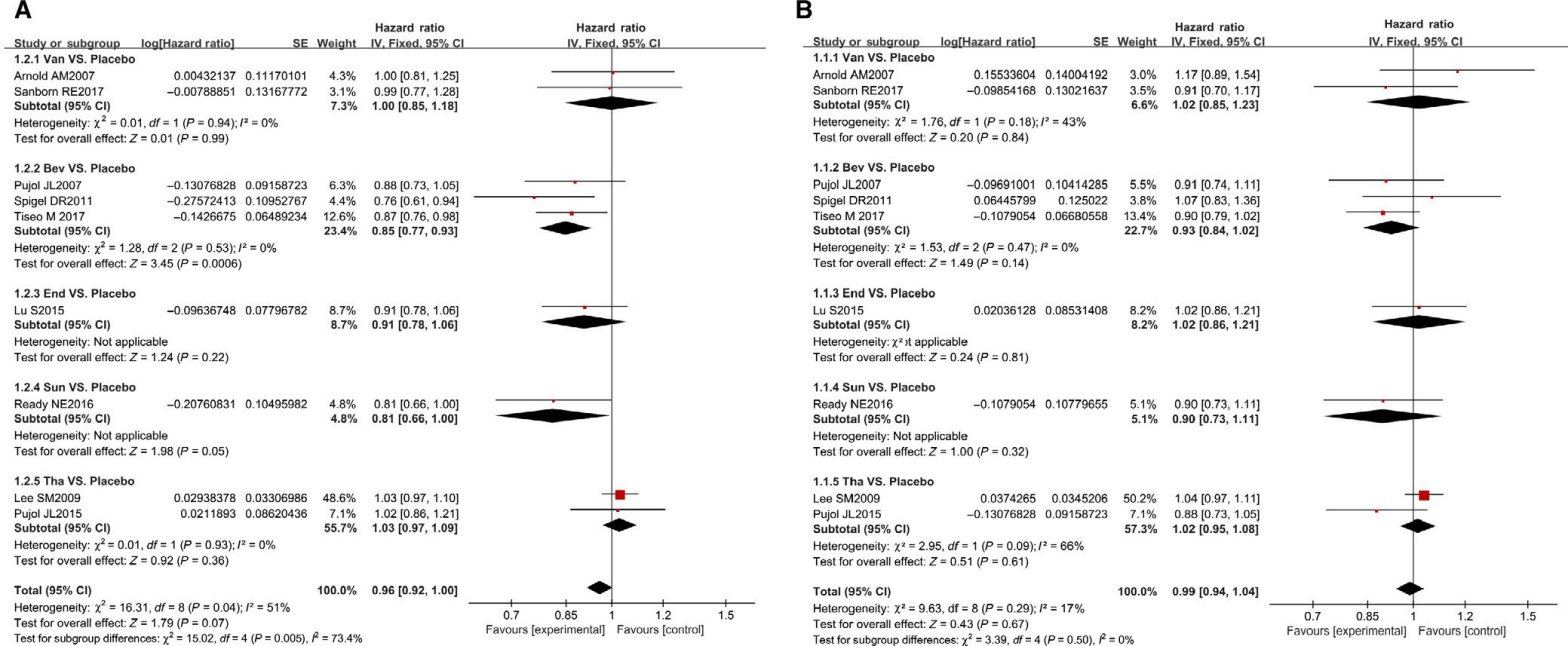
The survival meta‐analysis for angiogenesis inhibitors on SCLC. A, The merged PFS is reported by HR (95% CI). B, The merged OS is reported by HR (95% CI)

First, we conducted heterogeneity tests. In the meta‐analysis of direct comparison between two studies, there was significant heterogeneity based on the measurement of I^2^ = 66% and *P* = .09 in the OS of Tha VS. placebo, so the random effect model was applied. The remaining groups had no significant heterogeneity, so the fixed effect model was used. The results showed that there were significant differences between Bev VS. Placebo and Sun VS. Placebo in PFS (Bev VS. Placebo, HR = 0.85, 95%CI: 0.77‐0.93, Z = 3.45, *P* < .01; Sun VS. Placebo, HR = 0.81, 95%CI: 0.66‐1.00, Z = 1.98, *P* = .05). There was no significant PFS and OS differences among the other groups (Table S2 and Figure 2).

### 3.3 Network meta‐analysis

**Figure 3:**
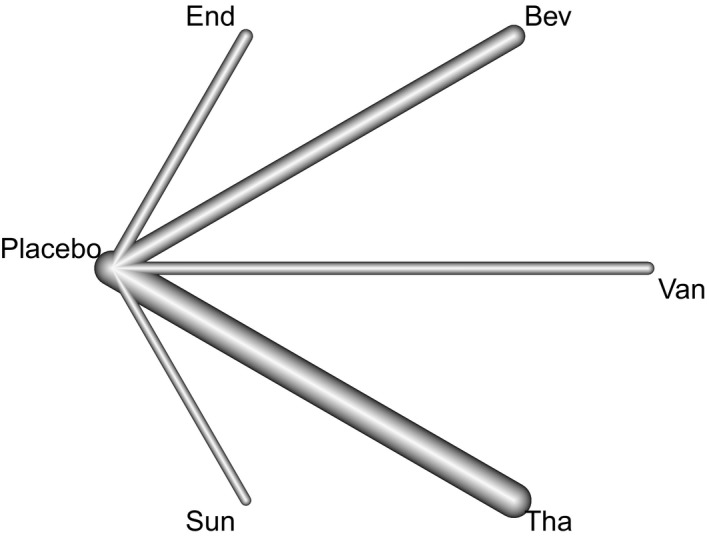
The treatment network. In terms of using placebo as control, the Van, Bev, Sun, Tha, and End are comparable, but no direct comparison between angiogenesis inhibitors was able to be analyzed

Firstly, a treatment network of PFS and OS from the same literature was constructed (Figure 3). It was found that Van, Bev, Sun, Tha, and End could be directly compared with placebo, but there was no direct comparison between angiogenesis inhibitors.

In terms of PFS, the internal and inter‐study heterogeneity was evaluated using Q‐statistics to construct the network meta‐analysis. The outcomes indicated that the fixed effect model could be applied (Table S3) The network meta‐analysis (Table 2) showed that Sun and Bev were better than Tha in terms of PFS. The PFS of Sun and Bev was significantly different from Tha (Bev VS. Tha, HR = 0.88, 95% CI: 0.79‐0.98, Sun VS. Tha, HR = 0.80, 95% CI: 0.65‐1.00), and that of Sun and Bev was significantly different from placebo (Bev VS. Placebo, HR = 0.89, 95% CI: 0.81‐0.97, Sun VS. Placebo, HR = 0.81, 95% CI: 0.66‐1.00). Sensitivity analysis was carried out by performing a random effect model and a fixed effect model on *P*‐score, respectively (Table S4). Based on the fact that the Funnel plot was basically symmetrical, publication bias was avoided in this work (Figure S1).

**Table 2:** The merged PFS and OS of network meta‐analysis

**Bev**	0.9167[0.7128;1.1787]	1.0421[0.7855;1.3826]	0.9114[0.8093;1.0264]	0.9090[0.7352;1.1239]	0.9266[0.8380;1.0246]
0.9749[0.7552;1.2585]	**End**	1.1369[0.8176;1.5808]	1.0039[0.8395;1.2004]	1.0012[0.7791;1.2866]	1.0206[0.8634;1.2063]
1.0896[0.8152;1.4564]	1.1177[0.7966;1.5681]	**Sun**	0.8830[0.7082;1.1009]	0.8807[0.6642;1.1677]	0.8977[0.7267;1.1089]
0.8785[0.7868;0.9809]	0.8983[0.7620;1.0589]	0.8037[0.6485;0.9960]	**Tha**	0.9973[0.8187;1.2149]	1.0166[0.9543;1.0831]
0.8889[0.7346;1.0754]	0.9088[0.7248;1.1397]	0.8132[0.6239;1.0598]	1.0118[0.8470;1.2086]	**Van**	1.0194[0.8456;1.2289]
0.8882[0.8102;0.9736]	0.9081[0.7794;1.0581]	0.8125[0.6614;0.9981]	1.0110[0.9512;1.0745]	0.9992[0.8456;1.1808]	**Placebo**

Note

The HRs (95% CI) are separated by abbreviations in bold, with the merged PFS result shown at the left lower corner and the merged OS shown at the right upper corner.^15^, ^16^, ^17^, ^18^, ^19^, ^20^, ^21^, ^22^, ^23^

Next, the internal heterogeneity and heterogeneity between the studies of OS were calculated by Q value (Table S5). According to the results of the network meta‐analysis (Table 2), the OS of Sun and Bev was better than Tha, but the difference of OS between the groups was not statistically significant. Sensitivity analysis showed that the top two rankings were consistent rather than the latter (Table S4). It was found that the funnel plot was basically symmetrical, indicating no publication bias (Figure S2).

### 3.4 Systemic downregulation of angiogenesis in SCLC RNA sequencing profile

**Figure 4:**
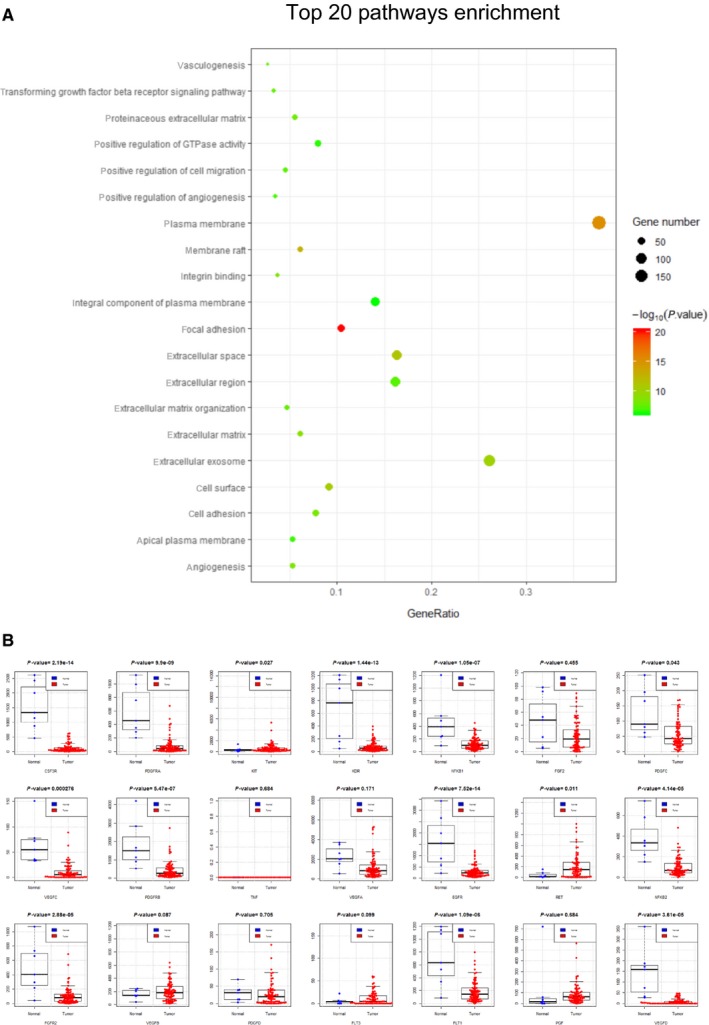
The angiogenesis‐associated genes and pathways are downregulated in SCLC tissues. A, Top 20 of 142 significantly downregulated signal pathways in SCLC tissue were showed, angiogenesis‐associated pathways are highlighted in red. B, In comparison to control tissue, the expression of angiogenesis inhibitor target genes was globally low expressed, indicating an inhibitory or nonactivated status of SCLC intratumor microenvironment

We further explored the underlying mechanisms of angiogenesis inhibitors in SCLC by bioinformatics analysis. The top 500 most significantly downregulated genes (Table S6) were selected for the gene ontology analysis. We found that the terms angiogenesis, transforming growth factor beta receptor signaling pathway, vasculogenesis, and positive regulation of angiogenesis enrichment were enriched (Figure 4A), suggesting that these biological processes were inactivated in SCLC tissue. Referred to the review literature^2^ and the drug instructions, we further analyzed the expression of target genes of angiogenesis inhibitors. As shown in Figure 4B, the results indicated that the expression of angiogenesis inhibitor targets genes, such as PDGFRA, PDGFRB, PDGFRC, VEGFC, VEGFD, and EGFR, was significantly downregulated in contrast to control tissue.

## 4 DISCUSSION

Compared with non‐small cell lung cancer, SCLC has a higher microvessel count, and this plays an important role in the metastasis process,^24^ so the angiogenesis inhibitor treatment of SCLC is feasible. The current clinical trials that were reported on antiangiogenic therapy for SCLC include: bevacizumab^22^, vandetanib,^21^ sunitinib,^20^ sorafenib,^25^ cediranib,^26^ nintedanib,^27^ aflibercept,^28^ endostar,^29^ thalidomide,^16^ and pomalidomide.^30^ However, most of the clinical trials of antiangiogenic therapy for SCLC in the past decade have ended in failure.^31^ For example, the SALUTE study indicated that the initial treatment of ED‐SCLC with Bev resulted in a significant prolongation of PFS (mPFS5.5 vs. 4.4 months) compared to the standard regimen, but no OS benefit was shown.^22^ In this study, the results of our meta‐analysis showed that Bev did have a significant impact on survival results.

The most studied antiangiogenic drug of SCLC is Bev. Several studies have indicated Bev as a first‐line treatment for SCLC, as it can prolong survival PFS,^22^, ^32^ but no OS improvement was shown^22^. Some studies also reported that combination use of Bev in the initial treatment of ED‐SCLC did not improve PFS.^19^ In contrast, the standard regimen combined with Bev maintenance therapy significantly prolonged PFS (5.7 vs. 6.7 months, *P* = 0.030).^23^ The outcome of our meta‐analysis suggests that Bev in the treatment of SCLC only improves PFS rather than OS.

Currently, there are no reports of Sun use for the first‐line treatment of SCLC. Sun has a 1‐year OS rate of 54% in maintenance therapy of ED‐SCLC.^33^ The clinical trial CALGB30504 indicated that Sun may improve PFS with an extension of 1.6 months.^20^ As PFS improvement was confirmed in our study, the insignificant OS improvement maybe due to the CALGB30504 study allowing the placebo group to also take Sun in the later stage.^20^ The therapeutic impact of Sun on ED‐SCLC survival requires further examination.

Our bioinformatics analysis aimed to exploit the large amount of SCLC sequencing data to unveil a possible mechanism for the incompetence of angiogenesis inhibitors on SCLC. The angiogenic pathway‐associated genes in SCLC tissues were globally low expressed, suggesting that angiogenic pathways are not activated in most cases of SCLC in the selected RNA sequencing data. At the risk of sample bias, our results imply that the angiogenesis activity may not be activated and keep a maintaining role for the growth of SCLC at different stage or at different part of tissue in the tumor. Future SCLC biomarker studies by sequential RNA sequencing or single‐cell sequencing for angiogenesis inhibitors should be done and the data should be publicly available.

### 4.1 Limitation

Our study is the first to analyze the overall efficacy of angiogenesis inhibitors in the treatment of SCLC by combining network meta‐analysis and bioinformatic analysis, providing evidence for further clinical practice. There were some limitations of our analysis: (a) only nine articles were available and the patients are mostly Caucasian patients from Europe and America, with only one study from China which may cause certain selection bias; (b) some sponsorship bias may exist; (c) it is impossible to conduct a comprehensive analysis of all indicators, for example side effects, due to the limited available data for different drug combinations; (d) all the analyzed studies were stage II‐III clinical trials, without phase IV clinical studies, so follow‐up update clinical trials are needed; (e) currently, few RNA sequencing data are publicly available for SCLC bioinformatic reanalysis, preventing a comprehensive analysis.

## 5 CONCLUSIONS

In conclusion, by an integrative evaluation of current clinical data about angiogenesis inhibitor and RNA expression profile of SCLC, Sun and Bev were the better options for use as angiogenesis inhibitors for SCLC. However, deeper understanding of the key biological function inside SCLC tissue could discover better target so as to improve therapeutic effect.

## ETHICS APPROVAL AND CONSENT TO PARTICIPATE

Not Applicable.

## CONSENT FOR PUBLICATION

All authors read and approved the publication of this manuscript.

## CONFLICT OF INTERESTS

The authors declare that they have no competing interests.

## AUTHOR CONTRIBUTIONS

Kaican Cai and Xiaoshun Shi conceived the idea as well as grant receiver of this project. Xiaoshun Shi, Xiaoying Dong, and Xiguang Liu performed studies selection and data extraction. Allen Menglin Chen, Kailing Huang, and Zhouxia Zheng performed RNA sequencing data extraction and bioinformatic analysis. Xiaoshun Shi performed network meta data analysis and interpretation of bioinformatic results. All authors participated in the manuscript preparation, proofreading, and submission.

## Data Availability

Not applicable.

## References

[1] Waqar SN , Morgensztern D . Treatment advances in small cell lung cancer (SCLC). Pharmacol Ther. 2017;180:16‐23. 28579387 10.1016/j.pharmthera.2017.06.002

[2] Stratigos M , Matikas A , Voutsina A , Mavroudis D , Georgoulias V . Targeting angiogenesis in small cell lung cancer. Transl Lung Cancer Res. 2016;5:389‐400. 27652203 10.21037/tlcr.2016.08.04 PMC5009078

[3] Srivastava R , Lebowicz Y , Jamil MO . Targeted agents in the management of small cell lung cancer ‐ present and future. Drugs Today (Barc). 2018;54:479‐488. 30209442 10.1358/dot.2018.54.8.2833977

[4] Ellis LM , Hicklin DJ . VEGF‐targeted therapy: mechanisms of anti‐tumour activity. Nat Rev Cancer. 2008;8:579‐591. 18596824 10.1038/nrc2403

[5] Fontanini G , Faviana P , Lucchi M , et al. A high vascular count and overexpression of vascular endothelial growth factor are associated with unfavourable prognosis in operated small cell lung carcinoma. Br J Cancer. 2002;86:558‐563. 11870537 10.1038/sj.bjc.6600130 PMC2375289

[6] Salven P , Ruotsalainen T , Mattson K , Joensuu H . High pre‐treatment serum level of vascular endothelial growth factor (VEGF) is associated with poor outcome in small‐cell lung cancer. Int J Cancer. 1998;79:144‐146. 9583728 10.1002/(sici)1097-0215(19980417)79:2<144::aid-ijc8>3.0.co;2-t

[7] Potente M , Gerhardt H , Carmeliet P . Basic and therapeutic aspects of angiogenesis. Cell. 2011;146:873‐887. 21925313 10.1016/j.cell.2011.08.039

[8] Ivy SP , Wick JY , Kaufman BM . An overview of small‐molecule inhibitors of VEGFR signaling. Nat Rev Clin Oncol. 2009;6:569‐579. 19736552 10.1038/nrclinonc.2009.130

[9] Jayson GC , Kerbel R , Ellis LM , Harris AL . Antiangiogenic therapy in oncology: current status and future directions. Lancet. 2016;388:518‐529. 26853587 10.1016/S0140-6736(15)01088-0

[10] Jiang L , Huang J , Higgs BW , et al. Genomic landscape survey identifies SRSF1 as a key oncodriver in small cell lung cancer. PLoS Genet. 2016;12:e1005895. 10.1371/journal.pgen.1005895 PMC4836692 27093186

[11] da Huang W , Sherman BT , Lempicki RA . Systematic and integrative analysis of large gene lists using DAVID bioinformatics resources. Nat Protoc. 2009;4:44‐57. 19131956 10.1038/nprot.2008.211

[12] da Huang W , Sherman BT , Lempicki RA . Bioinformatics enrichment tools: paths toward the comprehensive functional analysis of large gene lists. Nucleic Acids Res. 2009;37:1‐13. 19033363 10.1093/nar/gkn923 PMC2615629

[13] Higgins JP , Jackson D , Barrett JK , et al. Consistency and inconsistency in network meta‐analysis: concepts and models for multi‐arm studies. Res Synth Methods. 2012;3:98‐110. 26062084 10.1002/jrsm.1044 PMC4433772

[14] Rucker G , Schwarzer G . Ranking treatments in frequentist network meta‐analysis works without resampling methods. BMC Med Res Methodol. 2015;15:58. 26227148 10.1186/s12874-015-0060-8 PMC4521472

[15] Arnold AM , Seymour L , Smylie M , et al. Phase II study of vandetanib or placebo in small‐cell lung cancer patients after complete or partial response to induction chemotherapy with or without radiation therapy: national cancer institute of Canada clinical trials group study BR.20. J Clin Oncol. 2007;25:4278‐4284. 17878480 10.1200/JCO.2007.12.3083

[16] Lee SM , Woll PJ , Rudd R , et al. Anti‐angiogenic therapy using thalidomide combined with chemotherapy in small cell lung cancer: a randomized, double‐blind, placebo‐controlled trial. J Natl Cancer Inst. 2009;101:1049‐1057. 19608997 10.1093/jnci/djp200

[17] Lu S , Li LU , Luo YI , et al. A multicenter, open‐label, randomized phase II controlled study of rh‐endostatin (Endostar) in combination with chemotherapy in previously untreated extensive‐stage small‐cell lung cancer. J Thorac Oncol. 2015;10:206‐211. 25654728 10.1097/JTO.0000000000000343

[18] Pujol JL , Breton JL , Gervais R , et al. Phase III double‐blind, placebo‐controlled study of thalidomide in extensive‐disease small‐cell lung cancer after response to chemotherapy: an intergroup study FNCLCC cleo04 IFCT 00–01. J Clin Oncol. 2007;25:3945‐3951. 17761978 10.1200/JCO.2007.11.8109

[19] Pujol J‐L , Lavole A , Quoix E , et al. Randomized phase II‐III study of bevacizumab in combination with chemotherapy in previously untreated extensive small‐cell lung cancer: results from the IFCT‐0802 trialdagger. Ann Oncol. 2015;26:908‐914. 25688059 10.1093/annonc/mdv065

[20] Ready NE , Pang HH , Gu L , et al. Chemotherapy with or without maintenance sunitinib for untreated extensive‐stage small‐cell lung cancer: a randomized, double‐blind, placebo‐controlled phase II study‐CALGB 30504 (Alliance). J Clin Oncol. 2015;33:1660‐1665. 25732163 10.1200/JCO.2014.57.3105 PMC4429175

[21] Sanborn RE , Patel JD , Masters GA , et al. A randomized, double‐blind, phase 2 trial of platinum therapy plus etoposide with or without concurrent vandetanib (ZD6474) in patients with previously untreated extensive‐stage small cell lung cancer: Hoosier Cancer Research Network LUN06‐113. Cancer. 2017;123:303‐311. 27583688 10.1002/cncr.30287

[22] Spigel DR , Townley PM , Waterhouse DM , et al. Randomized phase II study of bevacizumab in combination with chemotherapy in previously untreated extensive‐stage small‐cell lung cancer: results from the SALUTE trial. J Clin Oncol. 2011;29:2215‐2222. 21502556 10.1200/JCO.2010.29.3423

[23] Tiseo M , Boni L , Ambrosio F , et al. Italian, multicenter, phase III, randomized study of cisplatin plus etoposide with or without bevacizumab as first‐line treatment in extensive‐disease small‐cell lung cancer: the GOIRC‐AIFA FARM6PMFJM trial. J Clin Oncol. 2017;35:1281‐1287. 28135143 10.1200/JCO.2016.69.4844

[24] Lucchi M , Mussi A , Fontanini G , Faviana P , Ribechini A , Angeletti CA . Small cell lung carcinoma (SCLC): the angiogenic phenomenon. Eur J Cardiothorac Surg. 2002;21:1105‐1110. 12048093 10.1016/s1010-7940(02)00112-4

[25] Gitlitz BJ , Moon J , Glisson BS , et al. Sorafenib in platinum‐treated patients with extensive stage small cell lung cancer: a southwest oncology group (SWOG 0435) phase II trial. J Thorac Oncol. 2010;5:1835‐1840. 20881645 10.1097/JTO.0b013e3181f0bd78 PMC3676180

[26] Ramalingam SS , Belani CP , Mack PC , et al. Phase II study of Cediranib (AZD 2171), an inhibitor of the vascular endothelial growth factor receptor, for second‐line therapy of small cell lung cancer (National cancer institute #7097). J Thorac Oncol. 2010;5:1279‐1284. 20559150 10.1097/JTO.0b013e3181e2fcb0 PMC2911495

[27] Han J‐Y , Kim HY , Lim KY , Hwangbo B , Lee JS . A phase II study of nintedanib in patients with relapsed small cell lung cancer. Lung Cancer. 2016;96:108‐112. 27133759 10.1016/j.lungcan.2016.04.002

[28] Allen JW , Moon J , Redman M , et al. Southwest oncology group S0802: a randomized, phase II trial of weekly topotecan with and without ziv‐aflibercept in patients with platinum‐treated small‐cell lung cancer. J Clin Oncol. 2014;32:2463‐2470. 25002722 10.1200/JCO.2013.51.4109 PMC4121504

[29] Zhou Z‐T , Zhou F‐X , Wei Q , Zou L‐Y , Qin B‐F , Peng X‐S . Phase II study of cisplatin/etoposide and endostar for extensive‐stage small‐cell lung cancer. Cancer Chemother Pharmacol. 2011;68:1027‐1032. 21327933 10.1007/s00280-011-1576-1

[30] Ellis PM , Jungnelius U , Zhang J , Fandi A , Beck R , Shepherd FA . A phase I study of pomalidomide (CC‐4047) in combination with cisplatin and etoposide in patients with extensive‐stage small‐cell lung cancer. J Thorac Oncol. 2013;8:423‐428. 23370364 10.1097/JTO.0b013e318282707b

[31] Li Q , Wu T , Jing LI , et al. Angiogenesis inhibitors for the treatment of small cell lung cancer (SCLC): A meta‐analysis of 7 randomized controlled trials. Medicine (Baltimore). 2017;96:e6412. 28353568 10.1097/MD.0000000000006412 PMC5380252

[32] Ready NE , Dudek AZ , Pang HH , et al. Cisplatin, irinotecan, and bevacizumab for untreated extensive‐stage small‐cell lung cancer: CALGB 30306, a phase II study. J Clin Oncol. 2011;29:4436‐4441. 21969504 10.1200/JCO.2011.35.6923 PMC3221525

[33] Spigel DR , Greco FA , Rubin MS , et al. Phase II study of maintenance sunitinib following irinotecan and carboplatin as first‐line treatment for patients with extensive‐stage small‐cell lung cancer. Lung Cancer. 2012;77:359‐364. 22560921 10.1016/j.lungcan.2012.03.009

