# Supplementary figures and images for: The impact of angiogenesis inhibitors on survival of patients with small cell lung cancer
Source: Cancer Med. 2019 Aug 21;8(13):5930–8. doi: 10.1002/cam4.2462 (PMC6792507; doi:10.1002/cam4.2462)

A

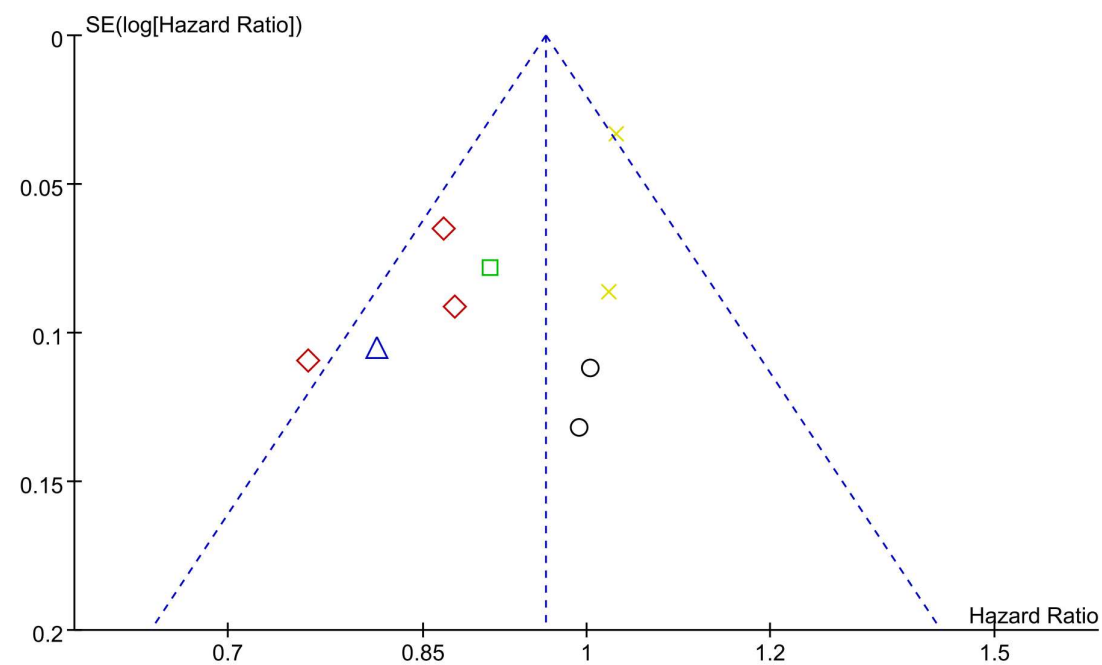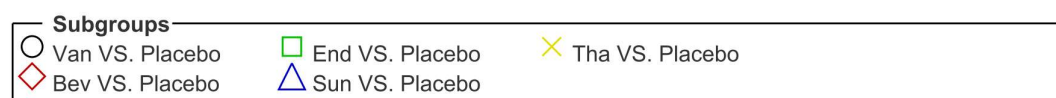

B

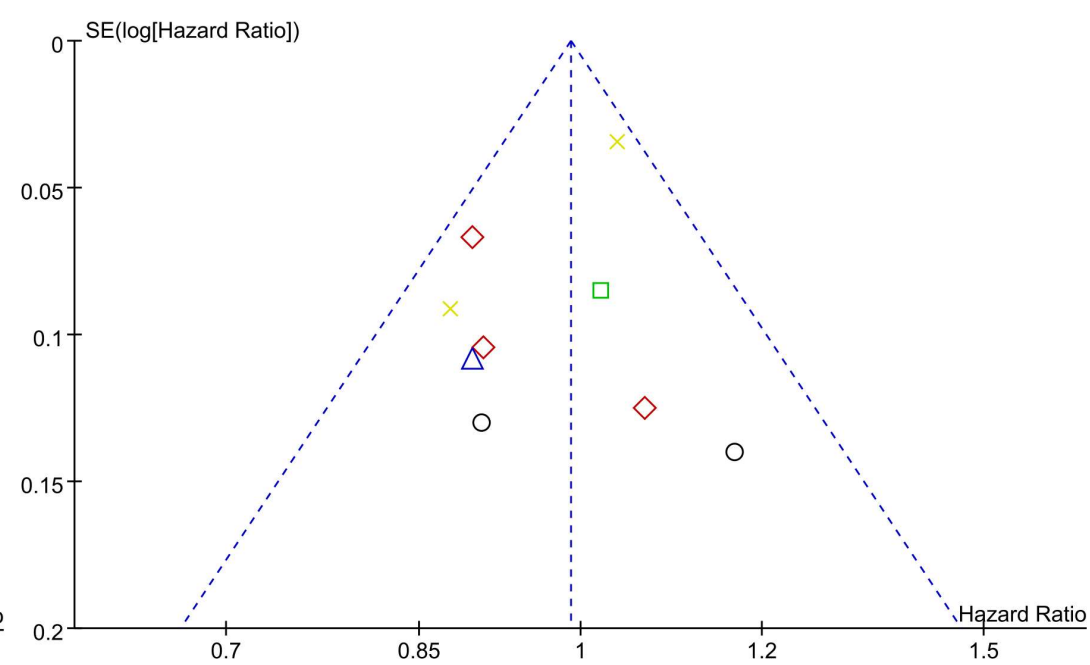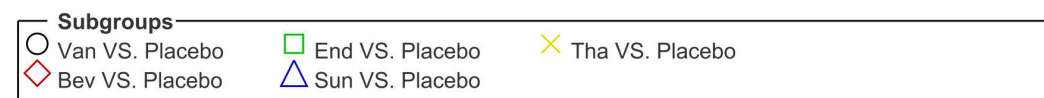

Supplement: Supplementary file 2 [file CAM4-8-5930-s002.pdf]
