# Supplementary material for: The impact of angiogenesis inhibitors on survival of patients with small cell lung cancer
Source: Cancer Med. 2019 Aug 21;8(13):5930–8. doi: 10.1002/cam4.2462 (PMC6792507; doi:10.1002/cam4.2462)
Supplement: Supplementary file 3 [file CAM4-8-5930-s003.docx]

**Table S1 Extraction of survival data**

| Author | Year | Group | Sample size | PFS(95% CI) | OS(95% CI) |
| --- | --- | --- | --- | --- | --- |
| Arnold AM | 2007 | Van | 53 | 1.01 (0.54-1.48) | 1.43 (0.63-2.23) |
|  |  | Placebo | 54 | NA | NA |
| Lee SM | 2009 | Tha | 365 | 1.07 ( 0.92-1.24) | 1.09 (0.93-1.27) |
|  |  | Placebo | 359 | NA | NA |
| Lu S | 2015 | End | 69 | 0.801(0.563-1.138) | 1.048(0.713-1.540) |
|  |  | Placebo | 69 | NA | NA |
| Pujol JL | 2007 | Tha | 49 | 0.74(0.49-1.12) | 0.74(0.49-1.12) |
|  |  | Placebo | 43 | NA | NA |
| Pujol JL | 2015 | Bev | 37 | 1.05(0.767-1.67) | 0.8(0.5-1.28) |
|  |  | Placebo | 37 | NA | NA |
| Ready NE | 2016 | Sun | 44 | 0.62(0.38-0.98) | 0.78(0.48-1.27) |
|  |  | Placebo | 41 | NA | NA |
| Sanborn RE | 2017 | Van | 40 | 0.982(0.542-1.779) | 0.797(0.443-1.435) |
|  |  | Placebo | 33 | NA | NA |
| Spigel DR | 2011 | Bev | 52 | 0.53(0.32-0.86) | 1.16 (0.66- 2.04) |
|  |  | Placebo | 50 | NA | NA |
| Tiseo M | 2017 | Bev | 95 | 0.72 (0.54- 0.97) | 0.78 (0.58-1.06) |
|  |  | Placebo | 103 | NA | NA |
