# Supplementary material for: The impact of angiogenesis inhibitors on survival of patients with small cell lung cancer
Source: Cancer Med. 2019 Aug 21;8(13):5930–8. doi: 10.1002/cam4.2462 (PMC6792507; doi:10.1002/cam4.2462)
Supplement: Supplementary file 4 [file CAM4-8-5930-s004.docx]

**Table S2 Meta-analysis of two groups**

|  | Groups | K | HR[95%CI] | Z | *P*_A_ | Model | Heterogeneity test | |
| --- | --- | --- | --- | --- | --- | --- | --- | --- |
|  |  |  |  |  |  |  | *P* | I^2^ (%) |
| PFS | Van VS. Placebo | 2 | 1.00 [0.85, 1.18] | 0.01 | 0.99 | Fixed | 0.94 | 0 |
|  | **Bev VS. Placebo** | **3** | **0.85 [0.77, 0.93]** | 3.45 | <0.01 | Fixed | 0.53 | 0 |
|  | End VS. Placebo | 1 | 0.91 [0.78, 1.06] | 1.24 | 0.22 | - | - | - |
|  | **Sun VS. Placebo** | **1** | **0.81 [0.66, 1.00]** | 1.98 | 0.05 | - | - | - |
|  | Tha VS. Placebo | 2 | 1.03 [0.97, 1.09] | 0.92 | 0.36 | Fixed | 0.93 | 0 |
| OS | Van VS. Placebo | 2 | 1.02 [0.85, 1.23] | 0.20 | 0.84 | Fixed | 0.18 | 43 |
|  | Bev VS. Placebo | 3 | 0.93 [0.84, 1.02] | 1.49 | 0.14 | Fixed | 0.47 | 0 |
|  | End VS. Placebo | 1 | 1.02 [0.86, 1.21] | 0.24 | 0.81 | - | - | - |
|  | Sun VS. Placebo | 1 | 0.90 [0.73, 1.11] | 1.00 | 0.32 | - | - | - |
|  | Tha VS. Placebo | 2 | 0.98 [0.83, 1.14] | 0.31 | 0.76 | Random | 0.09 | 66 |

*P_A_*: *P* value for test of the association.K: No. of studie; HR: hazard rate.
