# Supplementary material for: The impact of angiogenesis inhibitors on survival of patients with small cell lung cancer
Source: Cancer Med. 2019 Aug 21;8(13):5930–8. doi: 10.1002/cam4.2462 (PMC6792507; doi:10.1002/cam4.2462)
Supplement: Supplementary file 5 [file CAM4-8-5930-s005.docx]

**Supplement Table S3 The Heterogeneity test of PFS**

|  | Groups | Q | df | P |
| --- | --- | --- | --- | --- |
| Heterogeneity of Network meta-analysis | | 7.54 | 4 | 0.11 |
| Study internal heterogeneity | Placebo:Bev | 4.83 | 2 | 0.09 |
|  | Placebo:End | - | - | - |
|  | Placebo:Sun | - | - | - |
|  | Placebo:Tha | 2.71 | 1 | 0.10 |
|  | Placebo:Van | 0.01 | 1 | 0.94 |
| Inter-study heterogeneity | | - | - | - |
