# Supplementary material for: The impact of angiogenesis inhibitors on survival of patients with small cell lung cancer
Source: Cancer Med. 2019 Aug 21;8(13):5930–8. doi: 10.1002/cam4.2462 (PMC6792507; doi:10.1002/cam4.2462)
Supplement: Supplementary file 6 [file CAM4-8-5930-s006.docx]

**Table S4 P-score among groups**

| PFS | | | OS | | |
| --- | --- | --- | --- | --- | --- |
| Group | P-score | | Group | P-score | |
|  | fixed | random |  | fixed | random |
| Sun | 0.8947 | 0.8366 | Sun | 0.7895 | 0.7428 |
| Bev | 0.7374 | 0.7004 | Bev | 0.7818 | 0.6896 |
| End | 0.6376 | 0.5862 | Tha | 0.3059 | 0.4717 |
| Tha | 0.1892 | 0.3543 | Placebo | 0.4193 | 0.3838 |
| Van | 0.2869 | 0.2930 | End | 0.3453 | 0.3581 |
| Placebo | 0.2543 | 0.2294 | Van | 0.3582 | 0.3540 |
